# Presence of a Deletion Mutation of Myostatin (*MSTN*) Gene Associated With Double‐Muscling Phenotype in Japanese Black Cattle Population

**DOI:** 10.1111/asj.70055

**Published:** 2025-04-02

**Authors:** Nu Anh Thu Le, Rena Kubo, Liushiqi Borjigin, Takayuki Ibi, Shinji Sasaki, Tetsuo Kunieda

**Affiliations:** ^1^ Faculty of Veterinary Medicine Okayama University of Science Imabari Ehime Japan; ^2^ Faculty of Animal Science and Veterinary Medicine University of Agriculture and Forestry Hue University Hue Vietnam; ^3^ Graduate School of Environmental, Life, Natural Science and Technology Okayama University Okayama Japan; ^4^ Faculty of Agriculture Ryukyu University Nishihara Okinawa Japan

**Keywords:** double muscle, Japanese Black cattle, myostatin gene

## Abstract

Mutations in the bovine myostatin (*MSTN*) gene have been identified as the causative factor for the double‐muscling phenotype in several European cattle breeds, including Belgian Blue, Piedmontese, and Shorthorn. In Japan, following the Meiji Restoration, several European breeds, including Shorthorn, Brown Swiss, Devon, Simmental, and Ayrshire, were introduced and crossbred with native cattle to develop modern Japanese beef cattle breeds, such as Japanese Black cattle. Historical records regarding the breeding of Japanese Black cattle indicate that the double‐muscling phenotype, referred to as “Butajiri,” occasionally appeared in Japanese Black cattle population. These historical observations suggest the potential presence of *MSTN* gene mutation in the Japanese Black cattle population. The aim of this study was, therefore, to investigate the presence of *MSTN* gene mutation in the current Japanese Black cattle population. Through screening 400 reproductive females, we identified one cow carrying an 11‐bp deletion in the *MSTN* gene. While further investigation of the animals in the pedigree of this cow could not reveal any living animals with this mutation, this is the first report demonstrating the presence of the *MSTN* mutation in the Japanese Black cattle population.

## 1 Introduction

Myostatin (*MSTN*), also known as growth/differentiation Factor 8 or GDF‐8, is a key member of the transforming growth factor β (TGF‐β) family that regulates growth and differentiation of skeletal muscle cells. Inhibition of MSTN leads to muscular hypertrophy, characterized by significant increases in skeletal muscle mass and reduction in adipose tissue mass, as demonstrated in several domestic animals including cattle, sheep, goat, pigs, and dogs (Guo et al. 2009; Ren et al. 2020). The *MSTN* gene has, therefore, been widely regarded as a genetic target for improving meat production traits in domestic animals. In cattle, multiple mutations in the *MSTN* gene have been identified as the causative genetic factors for the double‐muscling phenotype in various breeds. These include an 11‐bp deletion in Exon 3 (c.818_828del), missense mutations (F94L and C313Y), and nonsense mutations that introduce premature termination codons (Q204X, E226X and E291X) (Grobet et al. 1997; Kambadur *et al*.,^1997^; McPherron and Lee 1997; Aiello, Patel, and Lasagna 2018). Among these, the 11‐bp deletion is the first identified *MSTN* mutation in cattle and the most prevalent mutation present in various beef cattle breeds including Angus, Belgian Blue, Piedmontese, South Devon, and Shorthorn (Aiello, Patel, and Lasagna 2018, Smith et al. 2000; Ryan et al. 2023, OMIA‐Online Mendelian Inheritance in Animals 2025).

In Japan, several European cattle breeds were introduced following the Meiji Restoration in the mid‐19^th^ century to meet the growing demands for meat and milk production. These breeds, including Shorthorn, Brown Swiss, Devon, Simmental and Ayrshire, were crossbred with native Japanese cattle to enhance their genetic potentials (Namikawa 1992). This led to the establishment of four Japanese original beef cattle breeds: Japanese Black, Japanese Brown, Japanese Shorthorn, and Japanese Polled. Among these, Japanese Black cattle (JBC) is the most predominant breed, known for producing highly marbled beef and accounting for 97% of total beef cattle in Japan (Gotoh et al. 2014, 2018; Hirooka 2014). Historical records for the breeding of JBC documented the occasional appearance of double‐muscling phenotype, referred to as “Butajiri” in the JBC population (Habe 1940; Sasaki 1994). This suggests the potential presence of *MSTN* gene mutation in the JBC population. However, to date, there have been no studies reporting the existence of *MSTN* mutations in JBC. Therefore, the aim of this study is to investigate whether *MSTN* mutation exists in the current JBC population. In the present study, we performed screening of the 11‐bp deletion of *MSTN* in JBC, since this mutation is the first identified *MSTN* mutation in cattle and is the most prevalent mutation present in various beef cattle breeds (OMIA‐Online Mendelian Inheritance in Animals 2025). The presence of this mutation in Japanese Shorthorn breed, which is established by cross between Japanese native cattle and Shorthorn breed (Muroya et al. 2009), also suggests that the 11‐bp deletion is the candidate mutation responsible for the Butajiri phenotype of JBC.

## 2 Materials and Methods

### 2.1 Collection and Extraction of Cattle DNA Samples

A total of 400 blood samples of JBC were obtained from reproductive females born between 1990 and 2009 on 19 farms in six prefectures across Japan. We also obtained blood samples of 21 animals in the pedigree of the cow that possesses the *MSTN* mutation (Figure 2). Genomic DNA was extracted from these blood samples using the standard phenol–chloroform extraction. Collection of blood samples was performed according to the guidelines for care and use of laboratory animals of Shirakawa Institute of Animal Genetics. The protocol was approved by the Shirakawa Institute of Animal Genetics Committee on Animal Research (H21–1).

### 2.2 Detection of the 11‐bp Deletion in the *MSTN* Gene by Amplicon Sequencing

To investigate the presence of the 11‐bp deletion in the *MSTN* gene within the JBC population, the 400 DNA samples were divided into four groups, each containing 100 samples. DNA concentration of all 400 samples was measured, and equimolar quantities (20 ng/μL/sample) from the 100 DNA samples in each group were pooled to create four pooled samples, designated as MG1, MG2, MG3, and MG4. A 241‐bp fragment encompassing the target region with the 11‐bp deletion was amplified from these pooled DNA using an adapter‐attached primer pair (listed in Table S1). PCR amplification was performed in a reaction mixture containing 10 ng of genomic DNA, 0.2 μM primers, and 1 U Kod FX *Taq* DNA polymerase (Toyobo, Osaka, Japan). The thermal cycling conditions included 30 cycles of denaturation at 94°C for 30 s, annealing at 62°C for 30 s, and extension at 72°C for 30 s. The amplified fragments were sequenced using amplicon sequence on an Illumina MiSeq NGS sequencer with read length of 2×300 bp.

### 2.3 Genotyping of the 11‐bp Deletion in the *MSTN* Gene by PCR and Cloning

Based on the results of amplicon sequencing, a sample group identified as positive for the 11‐bp deletion was selected and subjected to individual genotyping. To determine the genotype of the 11‐bp deletion in the *MSTN* gene, a 318‐bp fragment containing the deletion site was amplified from each DNA sample using the primer pair described in the Table S1. The PCR amplification was performed in the reaction mixture containing 10 ng of genomic DNA, 0.2 μM primers, and 1 U Kod FX *Taq* DNA polymerase (Toyobo, Osaka, Japan). The thermal cycling conditions included 30 cycles of denaturation at 94°C for 30 s, annealing at 58°C for 30 s, and extension at 72°C for 30 s. Amplified PCR products were purified by ExoSAP‐IT (Thermo Fisher) and sequenced via dideoxy termination method with the same primer pairs used in the PCR.

After identifying the heterozygous animal carrying the 11‐bp deletion, the amplified PCR product from the heterozygous animal was cloned into the pTA2 vector (Toyobo, Japan) and transformed into DH5α competent cells (Toyobo, Japan). Following incubation at 37°C for 17 h, positive colonies were selected. Their nucleotide sequences were determined by PCR and sequencing using the same primers and conditions described above.

### 2.4 Pedigree Investigation for the Animal Carrying the 11‐bp Deletion of the *MSTN* Gene

To identify additional animals carrying the 11‐bp deletion, the pedigree of the proband with the 11‐bp deletion was investigated using pedigree information (Figure 2). DNA samples were collected from the members of this pedigree, and their genotypes of *MSTN* gene were determined by direct sequencing of PCR product as described above.

## 3 Results

In the present study, we first performed the amplicon sequence of the target region containing the 11‐bp deletion in four pooled DNA samples (each composed of DNA from 100 individual JBC cows) to determine whether this mutation exists in the JBC population. The results revealed that the 11‐bp deletion in *MSTN* gene was detected at low frequency in one pooled DNA sample (MG1) with 290 mutant reads out of a total of 52,361 reads, while it was absent in the other three pooled DNA samples. The numbers of total, reference, and mutant reads are indicated in Table 1.

**TABLE 1:** Results of amplicon sequencing for *MSTN* gene.

Group	Total read number	Read number of reference sequence	Read number of 11‐bp deletion
MG1	52,361	52,071	290
MG2	34,914	34,914	0
MG3	33,202	33,202	0
MG4	42,025	42,025	0

**FIGURE 1:**
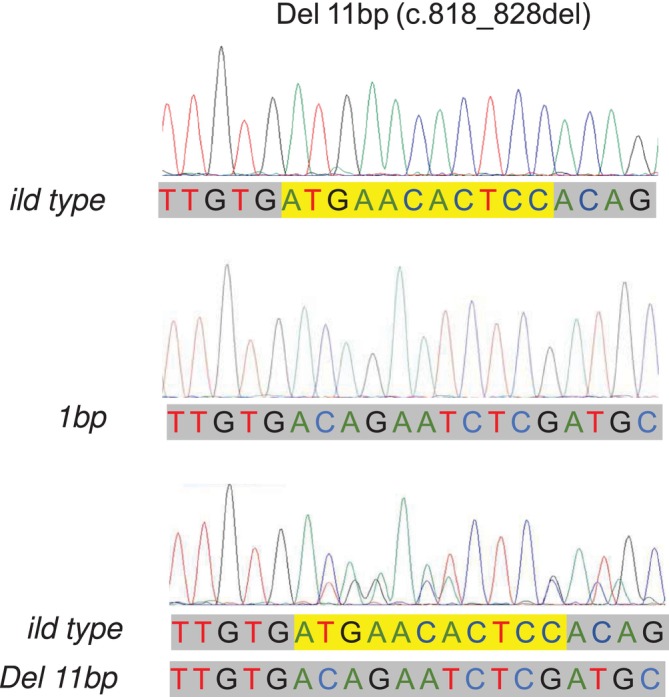
Detection of an 11‐bp deletion in *MSTN* gene in Japanese Black cattle. (A) Genomic structure of *MSTN* gene indicated by thick lines as exons and thin lines as introns and nucleotide sequence of a part of the amplified fragment including the 11‐bp deletion of *MSTN* gene. (B) Electropherograms of the region of *MSTN* gene including the 11‐bp deletion. (1) Wild type allele without the 11 bp‐deletion, (2) mutant allele with the 11‐bp deletion, and (3) hetrozygous animal carrying both wild type and mutant alleles.

These results suggested that animals carrying the 11 bp‐deletion were present in the 100 cows of MG1, but not in the other 300 cows. To identify individual animals carrying the 11‐bp deletion, we performed genotyping of the *MSTN* gene in all 100 animals of MG1 by PCR‐direct sequencing. As a result, we identified one animal with a heterozygous genotype for the 11‐bp deletion which showed two overlapping peaks in electropherogram of the Sanger sequence in the deleted region (Figure 1). All other animals were homozygous for the wild‐type allele without a deletion. The heterozygous genotype of the proband was confirmed by subcloning of the PCR products and sequencing of these clones. As shown in Figure 1, the clones from the proband exhibited the sequence lacking the 11‐bp region. These results clearly demonstrate that animals carrying an *MSTN* mutation are present in the current JBC population, albeit at a low frequency.

**FIGURE 2:**
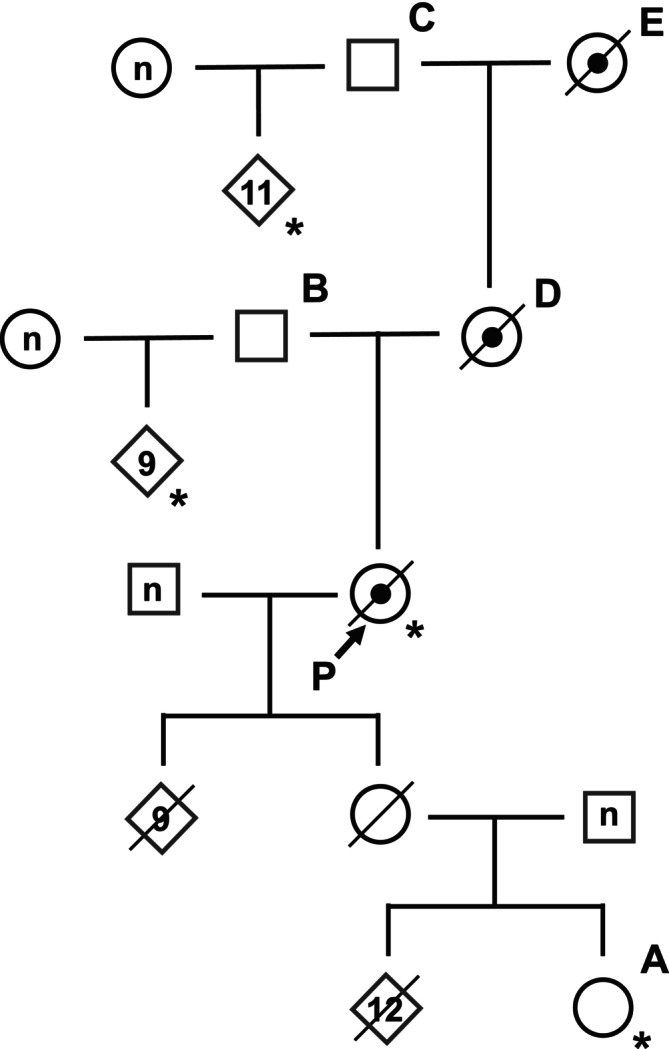
The pedigree tree of animals carrying the 11‐bp deletion of *MSTN* gene. Square: male; circle: female; rhombus: sex unknown; symbol with dot inside: heterozyogous carrier; symbol with a diagonal line: dead animal; number in symbol: number of animals at the same position in the pedigree tree; *n*: unknown number; arrow with P: proband; asterisk: animal with genotype confirmed by sequencing.

Unfortunately, the proband animal had already been slaughtered, and no living animal was available. To identify living animals carrying the mutant allele, we traced the inheritance of the mutant allele through her offspring. As shown in the pedigree tree (Figure 2), the proband produced 10 calves, and one of these offsprings produced 13 calves. However, we were unable to obtain DNA samples from any of these animals, except for one granddaughter (Animal A in Figure 2). Genotyping this living granddaughter revealed that she was homozygous for the wild‐type allele, indicating that she did not inherit the mutant allele from the proband. Next, we traced back to the ancestors of the proband. While we could not obtain DNA samples of her mother (D), father (B), maternal grandmother (E), and maternal grandfather (C), DNA samples were successfully obtained from 9 offspring of B and 11 offspring of C. The results of genotyping revealed that all 20 offspring of B and C were homozygous for the wild‐type allele, suggesting that both B and C did not carry the mutant allele. These results suggest that the mutant allele in the proband was inherited through the maternal line, likely from her mother (D) and maternal grandmother (E).

While our findings strongly suggest that D and E were carriers of the mutant allele, both animals had also been slaughtered, and we were unable to obtain their DNA samples to confirm their genotypes. Therefore, despite an extensive pedigree investigation spanning five generations, we could not identify any living animals carrying the *MSTN* mutant allele within the pedigree (Figure 2).

## 4 Discussion

The occurrence of cattle exhibiting the double‐muscle phenotype in JBC has been documented as “Butajiri” in historical records regarding JBC breeding (Habe 1940; Sasaki 1994). However, no studies have previously reported the existence of *MSTN* gene mutations in JBC. This study, therefore, represents the first evidence confirming the presence of the 11‐bp deletion in the *MSTN* gene within the JBC population. Given that the JBC breed was established through crossbreeding between Japanese native cattle and several European breeds, including Shorthorn (Namikawa 1992), and that Shorthorn breed is known to carry the 11‐bp deletion in the *MSTN* gene (Ryan et al. 2023), it is plausible that this mutation in JBC originated from the Shorthorn breed. Although only one heterozygous carrier was identified among the 400 animals screened, and this cow is no longer used for breeding, these findings suggest that the mutation may exist within the current JBC nationwide population, albeit at a low frequency. These results highlight the importance of large‐scale screening, monitoring, and management of the *MSTN* mutation in JBC, as its presence could have implications for breeding strategies as discussed below. In addition, it is to be noted that the mutations of *MSTN* other than the 11‐bp deletion, including F94L, C313Y, Q204X, E226X, and E291X, have been known to cause the double‐muscled phenotype in cattle. Therefore, there is a possibility that these mutations in addition to the 11‐bp deletion are also present in the JBC population, and further investigation of these mutations in JBC is necessary for comprehensive understanding of *MSTN* mutation in this breed.

There are dual effects of the *MSTN* mutations on the beef cattle production. While homozygous animals with the *MSTN* mutations show a markedly increased carcass weight due to muscular hypertrophy, the mutations of *MSTN* have been reported to negatively affect meat quality. For example, Allais et al. (2010) found that the animals carrying a nonsense mutation of *MSTN* exhibited carcasses with less intramuscular fat compared to wild‐type homozygous animals. Casas et al. (2004) also reported that animals with *MSTN* mutation showed lower beef marbling scores (BMSs) than those without the mutation. Considering these negative effects of *MSTN* mutation on meat quality, the mutations might lead to the undesirable traits for JBC including leaner and lower BMS. Indeed, “Butajiri” phenotype has been designated as one of the genetically defective traits that should be excluded from JBC by the Wagyu Registry Association (Wagyu Registry Association 2004).

The *MSTN* mutation is a genetically semidominant trait, wherein heterozygous animals exhibit intermediate phenotypes, such as slightly increased carcass weight (Gill et al. 2009; Ceccobelli et al. 2022; Allais et al. 2010). Due to the favorable traits associated with heterozygosity, there is a possibility of unintended selection of *MSTN* mutation carriers as sires. Given that large numbers of offspring are produced from a small number of sires through artificial insemination (AI), if a high‐performing AI sire carries undesirable mutations, the mutant allele can spread rapidly across the population. Some examples in the past highlight this risk, where heterozygous animals for specific genetic disorders were selected as sires due to their favorable traits associated with heterozygosity. Notable cases include tibial hemimelia in Shorthorn cattle (Whitlock, Kaiser, and Maxwell 2008) and skeletal dysplasia in JBC (Takasuga et al. 2015), where the frequency of mutant alleles increased by selecting carriers as sires, ultimately resulting in occurrence of many homozygous animals with the disorders. It is also noteworthy that the presence of a single gene with a large effect on a specific trait, such as *MSTN's* influence on carcass weight, can impact the accuracy of genomic selection. For instance, improved genomic prediction accuracy was reported when the effect of *MSTN* mutations was explicitly accounted for (Lee, Kim, and Garrick 2019), indicating the importance of accurately estimating the frequency and distribution of the *MSTN* mutations.

In conclusion, we identified one JBC cow with the 11‐bp deletion of *MSTN*, and this finding suggests the possibility of widespread presence of the mutation within the current population of JBC. Therefore, in the breeding of JBC, special attention will be necessary to prevent an increase in the frequency of the mutation and to avoid the occurrence of homozygous animals. Finally, we state that the limitation of this study is that we solely report the presence of the 11‐bp deletion in JBC breed and could not estimate the allele frequencies and the distribution of the *MSTN* mutation in JBC local populations due to the limited number of the samples, and therefore, a large‐scale screening across the various prefectural local populations of JBC is required in the future to assess the precise frequencies and distributions of the *MSTN* mutation.

## Conflicts of Interest

The authors declare no conflicts of interest.

## Supporting information


**Table S1** Primer sequence, amplified fragment size, and the position of 11‐bp deletion in the MSTN gene
